# Centroid Regression for Preoperative Risk Assessment of Acute Type A Aortic Dissection Based on Multivariate Clinical Data

**DOI:** 10.3390/jcm15135277

**Published:** 2026-07-06

**Authors:** Yiming Xiong, Zichun Tang, Yu Liu, Chen Lu, Yajing Li, Jia Hu, Xiaoyan Yang

**Affiliations:** 1 Department of Cardiovascular Surgery, West China Hospital, Sichuan University, Chengdu 610041, China2023151620332@stu.scu.edu.cn (Z.T.); 2023151620609@stu.scu.edu.cn (Y.L.); 2 West China Hospital/West China School of Medicine, Sichuan University, Chengdu 610041, China; 3 West China Biomedical Big Data Center, West China Hospital/West China School of Medicine, Sichuan University, Chengdu 610041, China

**Keywords:** centroid regression, acute type A aortic dissection, classification model, mortality risk

## Abstract

**Background/Objectives**: Acute type A aortic dissection (ATAAD) has high preoperative mortality, and an interpretable multivariable model based on clinically accessible data is crucial for clinical risk stratification. **Methods**: The data for this study were obtained from West China Hospital, Sichuan University. Centroid regression was used to construct the predictive model, with logistic regression, classification and regression tree, explainable boosting machine and extreme gradient boosting as the reference. Variables were screened by iterative selection, the literature review and clinical experience. Model performance was evaluated by accuracy, sensitivity, precision, Youden’s index, AUROC and AUPRC. **Results**: The vital signs and tests of 361 ATAAD patients during the first 24 h of their first admission were included in the final analysis. Centroid regression outperformed logistic regression, achieving accuracy (90.7% vs. 81.5%), sensitivity (0.813 vs. 0.741), specificity (0.983 vs. 0.900), Youden’s index (0.796 vs. 0.641), AUROC (area under the receiver operating characteristic curve, 0.953 vs. 0.843) and AUPRC (area under the precision–recall curve, 0.978 vs. 0.863) in the test set. It revealed that the use of α-blocker (the weights w = −1.20) and hydrochlorothiazide (w = −1.20), clinical features like dyspnea (w = −0.94), chest pain (w = 0.91) and lactate dehydrogenase (w = −0.95) were variables that had the greatest impact on model prediction. **Conclusions**: The centroid regression model not only has relatively high predictive performance and interpretability but also can be easily implemented in hospital systems to provide a practical and cost-effective tool for ATAAD preoperative risk stratification.

## 1. Introduction

Acute type A aortic dissection (ATAAD) is a critical condition associated with high mortality [1]. Timely diagnosis, accurate risk stratification, and prompt intervention are crucial, as mortality increases by up to 2% per hour following symptom onset [2]. Furthermore, patients residing in developing nations frequently face lengthy delays from symptom onset to definitive surgical intervention. In China, the interval between ATAAD symptom presentation and operative repair spans 2 to 7 days, in stark contrast to developed countries, where the average pre-surgical waiting time is only 4.5 h [3].

Advances have been made in surgical techniques, perioperative care, and postoperative management. Without standardized preoperative mortality stratification, clinicians may formulate inappropriate therapeutic plans and allocate medical resources unreasonably for high-risk ATAAD patients, which may delay optimal intervention; timely surgical intervention remains the core life-saving measure for such critical cases [4]. Furthermore, systematic risk scoring can mitigate subjective evaluation bias when identifying vulnerable individuals [5]. Although prior studies have evaluated the prognostic value of certain biomarkers in ATAAD patients, including cardiac troponin, D-dimer and brain natriuretic peptide [6,7,8], there is currently no reported study on multivariate predictive models for preoperative mortality in this population.

For a predictive model to be applicable in the urgent clinical setting of ATAAD, it must simultaneously possess high predictive accuracy, computational efficiency, and the trust of both clinicians and patients. The interpretability of the model is directly linked to the level of trust that users place in it [9]. However, there is an inherent trade-off between model accuracy and interpretability [10,11]. Black-box models often achieve superior predictive performance at the expense of interpretability, whereas linear models, despite being more transparent and interpretable, generally exhibit inferior predictive capability compared to nonlinear models [12,13,14].

Predictive models have been widely applied in the cardiovascular field [15,16]. Traditional clinical scoring systems deliver favorable predictive performance paired with robust interpretability, and mature research has validated their utility for risk and prognosis evaluation after ATAAD surgery; nevertheless, relevant evidence supporting their preoperative application remains absent [17,18]. The XGBoost algorithm achieves outstanding predictive capacity and enables identification of key risk factors, yet it suffers from limited interpretability, failing to quantify the magnitude of each predictor’s impact on mortality risk or delineate the changing trend of risk associated with each variable [19,20]. Most investigations focusing on clinical biomarkers only perform univariate analyses, which can clearly pinpoint single risk factors but result in severe underfitting when applied to aortic dissection cohorts [21,22]. Furthermore, Pan et al. constructed an ATAAD diagnostic model incorporating genetic data, with external validation conducted [23]. However, genetic testing suffers from low accessibility and prolonged turnaround time, restricting its practical deployment in time-sensitive emergency settings and medical facilities with limited resources.

Large-scale cohort studies focusing on preoperative mortality risk among ATAAD patients remain scarce, leading to insufficient standardized risk quantification for this population. Accordingly, there is an urgent demand for a preoperative mortality prediction tool tailored to patients from developing regions, which can facilitate early death risk identification and inform unified emergency management protocols across clinical centers. Our work aims to construct a streamlined, high-performance risk assessment model to quantify preoperative fatal risk based on 391 ATAAD patients presenting within 24 h of symptom onset.

## 2. Materials and Methods

### 2.1. Study Population and Definition

This study was designed as a retrospective cohort study. Patients diagnosed with acute type A aortic dissection (ATAAD) at West China Hospital, Sichuan University, between January 2010 and December 2020 were included in this study. The research was conducted in accordance with the Declaration of Helsinki (revised 2013) and approved by the Ethics Committee. Ethical approval was obtained from the Biomedical Ethics Committee of West China Hospital (No. 2026[212]).

Demographics, clinical and laboratory data, and relevant medical history of patients were collected upon admission. Patients who died before surgery were defined as positive samples, and the others were defined as negatives.

### 2.2. Model Derivation

#### 2.2.1. Data Pre-Processing

Patients with missing data were excluded. All samples were divided into training and test sets in a 7:3 ratio using a pseudo-random algorithm with equal probability.

Denote the number of samples as *n*, and the number of variables as *m*.

During data pre-processing for the model, each variable was standardized using its specific scaling function. Standardization involves proportionally scaling the output of the mapping function for each variable to the range [0, 1]. Each sample was transformed into an electric charge within an *m*-dimensional space [0, 1]*^m^*, where each dimension corresponds to the standardized value of one variable. The magnitude of the electric charge assigned to each sample is determined based on the number of negative and positive samples. Let the entire dataset be denoted as **D**, the outcome for each sample as *y* (*y* = 0 or 1), the corresponding position coordinates as **X** and the electric charge as *q*. The overall standardization process is as follows:

(1) $$q_{i}=\frac{y_{i}}{P_{n}}+\frac{y_{i}-1}{N_{n}}$$

(2) $$L_{j}=Min_{i=0}^{n-1}T_{j}\left(D_{i,j}\right),\;R_{j}=Max_{i=0}^{n-1}T_{j}\left(D_{i,j}\right),\;0\leq j<m$$

(3) $$X_{i,j}=\frac{D_{i,j}-L_{j}}{R_{j}-L_{j}},\;0\leq i<n,\;0\leq j<m$$

(4) $$T_{v}(x)=\left\{\begin{array}{c} x>60,\;v=<AST,ALT>\\ x>360,\;v=<LDH>\\ x,\;v=<OTHERS> \end{array}\right.$$

Denote *P_n_* as the number of positive samples and *N_n_* as the number of negative samples in Formula (1). Function *T* in Formulas (2) and (4) is the transforming function, and the assignment of *T* is associated with the variable. In Formula (4), *v* represents the name of the variable (AST: aspartate aminotransferase, ALT: alanine aminotransferase, LDH: lactate dehydrogenase).

#### 2.2.2. Variable Selection

**Figure 1:**
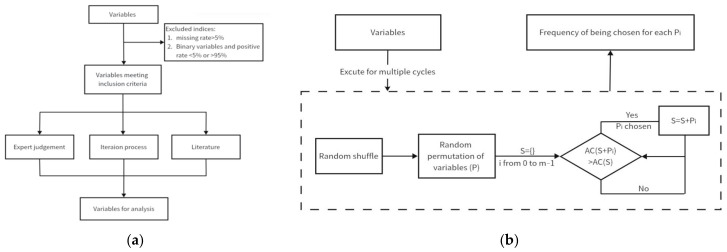
Flowchart of variable selection of centroid regression. (**a**) The general process of variable selection; (**b**) the iterative self-selection process in variable selection. In each cycle, the iteration starts with an empty set S (S = {}). The operator ‘+’ denotes the set with an element addition.

Denote AC(S) as the model’s accuracy when using the variable combination S. The iterative variable screening process repeatedly follows these steps, recording the frequency with which each variable is selected (shown in Figure 1):

1. The order of all variables is randomly shuffled, and an initially empty set S for selected variables is defined.
2. Each variable *v* is examined sequentially in the order generated in step 1. If the model’s accuracy with the set S + *v* is higher than that with S alone, then *v* is added to S.
3. Step 2 is repeated until all variables have been examined.
4. Count the variables in S. Variables in set S are selected variables in the iteration process. Then, the iteration ends.

Repeat the iteration (as steps 1–4 shown) and count the total frequency of each variable.

A higher frequency of a variable being selected into set S indicates a stronger potential correlation with ATAAD mortality. The variables would be excluded when the variable has no contribution to the prediction, or the accuracy of the model would not be lower if the variable is excluded.

The inclusion and exclusion criteria were as follows:

1. Exclude variables with a missing data rate > 5%.

2. Exclude binary variables with a positive rate < 5% or >95%.

3. Include variables with literature support.

4. Include variables with clinical expert support.

5. Include variables selected by the self-iterative process of the model with the top 10 selective frequencies.

6. Exclude variables that have no contribution to the accuracy of the model. The variable *v* would be excluded in the variable selection set S + *v* if AC(S + *v*) ≥ AC(S).

Subsequently, the samples were cleaned by excluding any sample that was missing one or more of the final selected variables (shown in Section 3.1), resulting in the final sample set available for model development.

#### 2.2.3. Training

Prior to training, data pre-processing has been completed, yielding the spatial coordinates and electric charge for each transformed sample.

The characteristic point or the centroid of the dataset is determined through the electric field iterative algorithm. The training objective of this algorithm is to locate the point with the highest electric potential within the field as the final result.

To obtain this point, we first use a pseudo-random algorithm to randomly and uniformly select an initial coordinate within the [0, 1]*^m^* space as the starting point for training (**P**_0_). A test charge is placed at this coordinate, with an initial step size (*δ*_0_ < $\sqrt{m}$) and a termination step size (*ε*) set, after which iterative updates commence. Each iteration calculates the electric field force acting on the test charge and updates its new position, while the step size is reduced.

Let **F** denote the electric field force acting on the test charge, *δ* represent the iteration step size, *κ* (0 < *κ* < 1) be the step size reduction coefficient, and *n* be the total number of samples in the training set. The *i*-th iteration proceeds as follows:

(5) $$F_{i}=\sum_{j=0}^{n-1}q_{j}(X_{j}-P_{i-1})|X_{j}-P_{i-1}{|}^{-3}$$

(6) $$P_{i}=P_{i-1}+\delta_{i-1}F_{i}|F_{i}{|}^{-1}$$

(7) $$\delta_{i}=\kappa \delta_{i-1}$$

**Figure 2:**
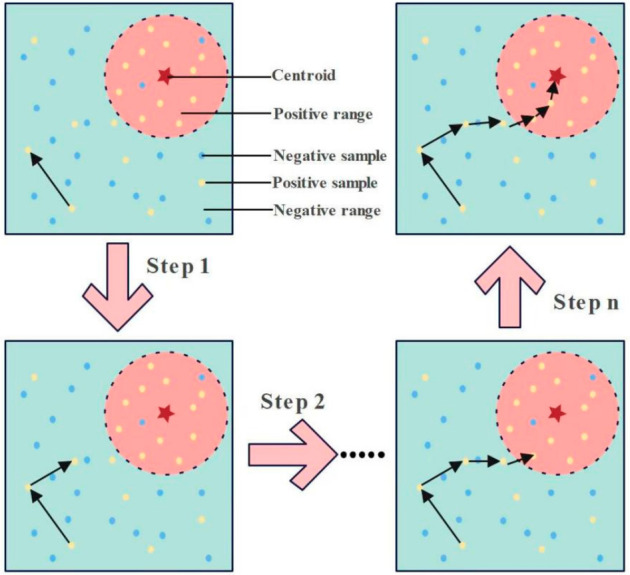
Schematic diagram of the centroid regression electric field iteration process. Each arrow in the figure represents the position change of **P** in a single iteration process.

The iteration terminates when the current step size is less than the termination step size (*δ* < *ε*), and the current point **P** is taken as the final result of the training. The illustration of the iteration process is shown in Figure 2.

#### 2.2.4. Testing

The Euclidean distance between the sample point and the centroid is used as the feature value. If a sample’s feature value is lower than the cut-off value, it is categorized as positive; otherwise, it is classified as negative.

Before calculating the feature values, the test set data must be processed using the same standardization functions applied during the pre-processing phase. Since these standardization functions do not depend on the numerical distribution of the test set data, a small number of standardized values may fall outside the [0, 1] range.

Let **X** represent the transformed coordinates of a sample and **C** denote the data centroid obtained from training. The feature value *d* is then expressed as

(8) $$d=(X-C{)}^{2}$$

The value *d* could also represent the mortality risk of an ATAAD patient.

The complexity and the interpretability are proved in Appendix A.

#### 2.2.5. Model Evaluation

Model performance was evaluated using accuracy, sensitivity, specificity, Youden’s index, AUROC (area under the receiver operating characteristic curve), and AUPRC (area under the precision–recall curve).

Using identical sample groupings and the exact same set of variables, logistic regression was applied to classify the samples, and the classification results of the two models were compared.

Classification and regression tree (CART), explainable boosting machine (EBM) and extreme gradient boosting (XGBoost) were also applied as the reference to assess the performance of centroid regression.

To further assess model robustness, K-fold cross-validation was performed (*k* = 10). The dataset was randomly partitioned into 10 folds of approximately equal size. Performance metrics across the 10 folds were reported as mean ± standard deviation.

### 2.3. Statistics Analysis

To investigate the homogeneity between the training and test sets, as well as the differences between positive and negative samples, we conducted the following comparative analyses: specifically, categorical variables were compared using the chi-square test; for continuous variables, comparisons were performed based on their distribution characteristics—employing the independent samples *t*-test (for normally distributed data) or the Mann–Whitney U rank-sum test (for non-normally distributed data). This part of the data analysis was completed using IBM SPSS Statistics (Version 27), with the significance level set at *α* = 0.05.

## 3. Results

### 3.1. Data Collection and Variable Selection

A total of 394 adult patients were initially included, comprising 233 preoperative deaths and 161 patients who survived and underwent surgery. Based on the inclusion and exclusion criteria, 33 patients with missing data were excluded, resulting in 361 patients included in the final analysis.

The samples with data missing are shown in Table 1, including samples that only lack medical history (≥5 variables missing), samples that lack laboratory tests (5 variables missing), the sample that lack coagulation action test and medical history (fibrinogen and hypertension missing) and samples that only lack liver and kidney function test (7 variables missing).

**Figure 3:**
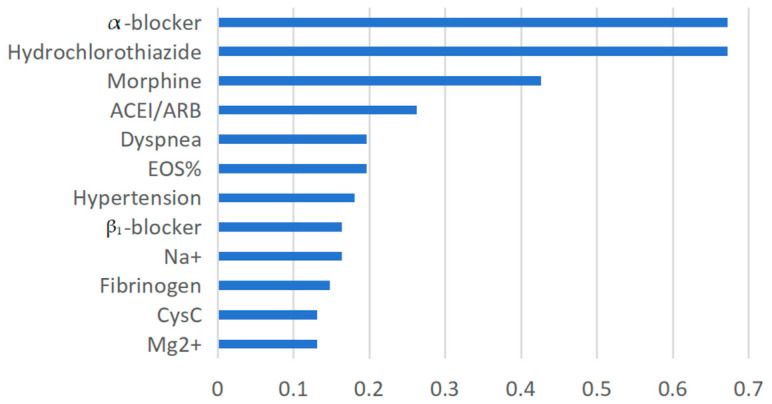
The twelve most frequently selected variables and their selection frequencies in the iterative variable screening process of the model.

During the iterative variable selection process of the model, the five variables selected most frequently were dyspnea, use of α-blockers, use of morphine, use of hydrochlorothiazide, and eosinophil percentage. The selection frequencies for some variables are shown in Figure 3.

The final 25 variables were selected and applied in the model development. The binary variables were: gender (male = 0), chest pain, back pain, abdominal pain, dyspnea, hypertension, the use of β_1_-blocker, ACEI/ARB (angiotensin-converting enzyme inhibitors/angiotensin II receptor blockers), α-blocker, morphine and hydrochlorothiazide, and the laboratory test of ALT (ALT > 60 U/L), AST (AST > 60 U/L) and LDH (LDH > 360 U/L). The continuous variables were: age, McV (mean corpuscular volume, fL), RDW-SD (red cell distribution width–standard deviation, fL), NEUT% (neutrophil percentage), LY% (lymphocyte percentage), EOS% (eosinophil percentage), fibrinogen, TBil (total bilirubin, μmol/L), CysC (Cystatin C, mg/L), Ca^2+^ (serum calcium, mmol/L) and Mg^2+^ (blood magnesium, mmol/L).

The 361 patients were randomly allocated, with 70% assigned to the training group and 30% to the test group, and 57.9% samples were positive. The mean age of the included patients was 54 years (SD = 12.7), 21.9% were female, and 44.3% had hypertension. The descriptive statistics are presented in Table 2.

The training set consisted of 253 patients and the test set of 108 patients. No statistically significant differences were observed in baseline characteristics and preoperative data between the two groups, as shown in Table 3. The overall distribution of variables was balanced across the training and test sets.

### 3.2. Training Result

After iterative computation in the electric field, the data centroid and weights for the binary variables were obtained, as shown in Table 4. The data centroid for continuous variables are presented in Table 5.

**Figure 4:**
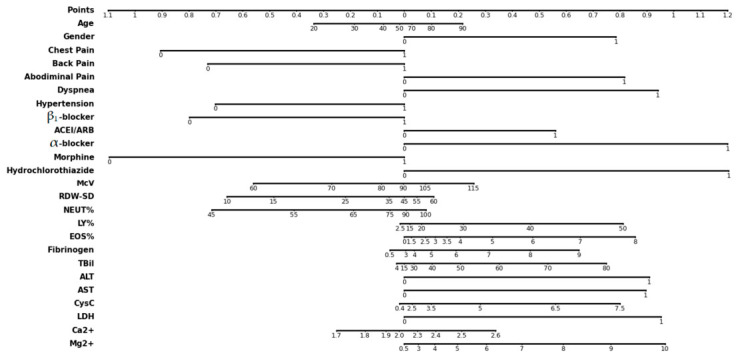
Nomogram of variables in the centroid regression model.

A nomogram was used to visually demonstrate the weight contribution of all 25 indicators in predicting patient mortality, which aids clinicians in risk stratification of patients, as illustrated in Figure 4.

### 3.3. Testing Result and Model Evaluation

**Figure 5:**
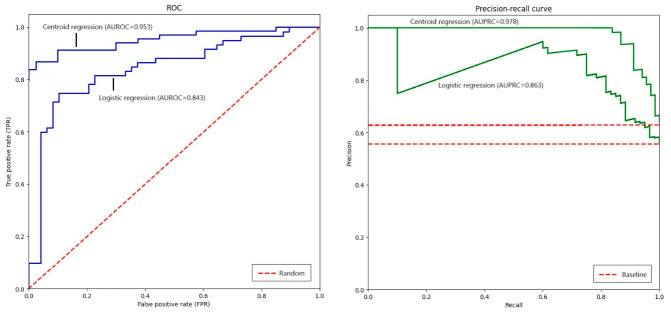
ROC and PRC curves for the centroid regression and logistic regression test results.

The confusion matrix of the test result of centroid regression is shown in Table 6, the prediction comparison between centroid regression and other models is shown in Table 7, and the ROC and PRC curves for the centroid regression and logistic regression test results are shown in Figure 5. Centroid regression, achieving an accuracy of 90.7%, a sensitivity of 0.813, a specificity of 0.983, Youden’s index of 0.796, AUROC of 0.953 and AUPRC of 0.978 in the test set, all outperformed logistic regression.

### 3.4. K-Fold Result

The dataset was randomly split into 10 sets, 9 sets with 36 samples and 1 set with 37 samples. The result of the K-fold was shown in Table 8.

The single test results (shown in Table 7) for accuracy, Youden’s index, AUROC, and AUPRC all fell within their respective 95% confidence intervals (CIs). However, sensitivity fell below the lower limit of its 95% CI, while specificity exceeded the upper limit of its 95% CI.

## 4. Discussion

Our high-impact predictors, including dyspnea, antihypertensive drug use and LDH, correspond to well-documented aortic histopathological changes [24]. Severe medial degeneration with elastic fiber loss and mucoid accumulation constitutes the core substrate of fatal ATAAD; uncontrolled hypertension worsens aortic wall shear stress [25], while dyspnea and elevated LDH act as clinical surrogates for tamponade [26], malperfusion and tissue necrosis, giving clear biological rationale to our model’s variable weights [27].

We further emphasize a critical interpretative distinction between predictive correlation and causal inference for medication-related predictors identified in our model, including α-blockers, hydrochlorothiazide and morphine [28]. The strong predictive weights of these drugs do not establish direct causal effects on preoperative mortality; instead, medication administration largely acts as a clinical surrogate reflecting baseline disease severity and emergency treatment patterns [29]. Morphine is selectively given to patients with intractable severe pain and heightened sympathetic activation, markers of advanced dissection injury. α-blockers and thiazide use reflect chronic long-term hypertension status and emergency hemodynamic stabilization efforts, rather than independently fatal biological drivers. Our centroid regression model is built exclusively for risk stratification based on observational admission data, which cannot disentangle confounding, reverse causation or treatment indication bias inherent to retrospective cohorts [30]. The parameters derived from the model should not be interpreted as etiological evidence. However, this does not compromise the model’s utility for risk prediction in real-world clinical settings.

Beyond biological context, clinically, this lightweight algorithm fills gaps in routine emergency management [30]. It only requires standard 24 h admission data with minimal computing overhead, enabling seamless integration into hospital electronic medical records. For developing regions with prolonged pre-surgical waiting intervals, the model standardizes triage stratification, mitigates subjective clinical bias, prioritizes high-risk patients for urgent surgery consultation, accelerates consultation for high-risk patients and rationalizes limited ICU and operating room resources [31]. Its fully interpretable variable gradients also support individualized preoperative hemodynamic adjustment at bedside, translating algorithmic performance into actionable emergency decision-making [32].

Clinical entities exhibit distinct pathophysiological characteristics and heterogeneous data distributions, and prior studies have confirmed that no single predictive model performs universally well across all disease spectra [33]. Conventional linear models are often inadequate to capture complex nonlinear patterns inherent in critical care data [34], while a one-size-fits-all strategy ignores disease specificity and leads to degraded performance and poor generalizability [35]. The centroid regression algorithm we proposed herein demonstrates clear superiority in adaptive screening of nonlinear data distributions via its electric field-based iterative framework. Given the unique data structure and specificity of each disease, risk prediction and classification should employ disease-tailored models rather than a generalized approach, which further underscores the innovation and clinical adaptability of centroid regression in handling heterogeneous and nonlinear clinical data.

Centroid regression demonstrated significantly better predictive performance than logistic regression, while exhibiting no significant differences in performance compared with CART or EBM, since the accuracy and AUROC of the two models were included in 95% CIs (shown in Table 7 and Table 8). Among all models evaluated, XGBoost achieved the highest performance. These findings suggest that the performance of centroid regression is comparable to that of more mature white-box models such as CART and EBM. Accordingly, further enhancement of predictive accuracy may be an important direction for model development. The robustness of the model has also been validated. The model shows relatively low sensitivity but high specificity, indicating that the cutoff value can be adjusted according to the specific clinical scenario.

We excluded 8.4% samples because of data missing. Missing data may be attributed to patients’ impaired consciousness at admission, which hindered history collection, and to non-attendance of scheduled examinations, leading to missing laboratory values. We have not conducted imputation methods, because all samples with data missing involved multiple variables and imputing data can not be determined to a unique value (Formula (8)). The value **X**_j_ = **C**_j_ + *v* could be the imputed value if **X**_j_ = **C**_j_ − *v* is the imputed value and all the missing data exists two potential imputed values.

The model exhibits a low risk of overfitting, supported by the 10-fold cross-validation results. Moreover, the model is fully transparent, and the number of parameters in the model is about equal to the number of predictors, ensuring low model complexity. Although this study is based on a single center with a relatively modest sample size, we consecutively enrolled all eligible patients during the study period.

## 5. Limitations

This retrospective single-center study has multiple inherent limitations. Constrained by ATAAD’s low incidence and domestic inter-hospital transfers, our limited cohort lacks external multi-center validation, restricting generalizability to populations with distinct medical backgrounds. Potential selection bias exists as we excluded patients with multi-item missing data rather than imputation, since centroid regression’s symmetric risk function cannot generate unique imputed values, possibly excluding unconscious critically ill individuals. Moreover, we only adopted 24 h admission routine indicators without long-term follow-up or aortic histopathological markers. The model was built solely on Chinese patients with delayed surgery. Thus, prospective multi-center studies are needed to externally verify and optimize this model.

## 6. Conclusions

The centroid regression approach we proposed offers a robust, intuitive and computationally efficient solution for preoperative risk evaluation in ATAAD, with distinctive strengths in modeling intricate clinical data patterns and delivering transparent decision support. Owing to its flexible structure and compatibility with routine clinical indicators, this method could be readily generalized to diverse prognostic tasks across various critical illnesses, helping clinicians implement prompt risk stratification, refine clinical judgments and enhance the delivery of personalized care, thereby bringing meaningful value to modern clinical practice and intelligent healthcare decision-making.

## Figures and Tables

**Table 1:** Descriptive of missing data.

	Positive Samples	Negative Samples	Total
Lack of medical history	17	4	21
Lack of laboratory test	2	2	4
Lack of coagulation action test and medical history	0	1	1
Lack of liver and kidney function tests	5	2	7
Total	24	9	33

**Table 2:** Descriptive statistics of 25 variables.

Variables	Total (n = 361)	Death (n = 209)	Survive (n = 152)	*p*-Value
Demographics				
Age, Mean ± SD	54.0 ± 12.7	55.7 ± 11.8	50.7 ± 13.4	<0.001
Gender, n (%)	79 (21.9)	43 (20.6)	36 (23.7)	0.520
Medical history				
Chest pain, n (%)	269 (74.5)	166 (79.4)	103 (67.8)	0.014
Back pain, n (%)	152 (42.1)	119 (56.9)	33 (21.7)	<0.001
Abdominal pain, n (%)	90 (24.9)	44 (21.1)	46 (30.3)	0.049
Dyspnea, n (%)	121 (33.5)	39 (18.7)	82 (53.9)	<0.001
Hypertension, n (%)	160 (44.3)	131 (62.7)	29 (19.1)	<0.001
β_1_-blocker, n (%)	238 (65.9)	160 (76.6)	78 (51.3)	<0.001
ACEI/ARB, n (%)	240 (66.5)	94 (45.0)	146 (90.1)	<0.001
α-blocker, n (%)	144 (39.9)	12 (5.7)	132 (86.8)	<0.001
Morphine, n (%)	189 (52.4)	171 (81.8)	18 (11.8)	<0.001
Hydrochlorothiazide, n (%)	150 (41.6)	19 (9.1)	131 (86.2)	<0.001
Laboratory test				
McV, Median (IQR)	91.8 (88.8, 94.7)	92.2 (89.5, 95.5)	91.0 (88.3, 94.1)	0.010
RDW-SD, Median (IQR)	44.4 (41.9, 46.8)	45.0 (42.2, 47.1)	43.9 (41.4, 46.6)	0.226
NEUT%, Median (IQR)	85.7 (80.7, 89.7)	86.5 (81.0, 90.0)	85.1 (80.5, 89.6)	0.320
LY%, Median (IQR)	7.7 (5.4, 11.2)	7.7 (5.2, 11.2)	8.1 (5.6, 11.2)	0.541
EOS%, Median (IQR)	0.1 (0.0, 0.3)	0.1 (0.0, 0.2)	0.1 (0.0, 0.4)	0.001
Fibrinogen, Median (IQR)	2.16 (1.61, 3.26)	1.99 (1.46, 2.84)	2.44 (1.75, 3.69)	<0.001
TBil, Median (IQR)	16.6 (11.6, 22.7)	16.6 (11.9, 22.8)	16.7 (11.1, 22.3)	0.904
CysC, Median (IQR)	1.03 (0.83, 1.39)	1.08 (0.85, 1.47)	0.94 (0.80, 1.23)	0.017
Ca^2+^, Median (IQR)	2.15 (2.06, 2.24)	2.14 (2.06, 2.22)	2.17 (2.10, 2.26)	0.007
Mg^2+^, Median (IQR)	0.82 (0.77, 0.90)	0.82 (0.77, 0.90)	0.81 (0.76, 0.89)	0.743
ALT, n (%)	50 (13.9)	32 (15.3)	18 (11.8)	0.360
AST, n (%)	61 (16.9)	42 (20.1)	19 (12.5)	0.065
LDH, n (%)	49 (13.6)	33 (15.8)	16 (10.5)	0.164

**Table 3:** Homogeneity assessment of training set and test set.

Variables	Training Set (n = 253)	Test Set (n = 108)	*p*-Value
Demographics			
Age, Mean ± SD	53.7 ± 12.8	53.4 ± 12.5	0.865
Gender, n (%)	52 (20.6)	27 (25.0)	0.404
Medical history			
Chest pain, n (%)	187 (73.9)	82 (75.9)	0.792
Back pain, n (%)	99 (39.1)	53 (49.1)	0.082
Abdominal pain, n (%)	64 (25.3)	26 (24.1)	0.894
Dyspnea, n (%)	88 (34.8)	33 (30.6)	0.467
Hypertension, n (%)	111 (43.9)	49 (45.4)	0.818
β_1_-blocker, n (%)	164 (64.8)	74 (68.5)	0.545
ACEI/ARB, n (%)	174 (68.8)	66 (61.1)	0.181
α-blocker, n (%)	105 (41.5)	39 (36.1)	0.350
Morphine, n (%)	125 (49.4)	64 (59.3)	0.107
Hydrochlorothiazide, n (%)	106 (41.9)	44 (40.7)	0.907
Laboratory test			
McV, Median (IQR)	91.8 (88.7, 95.0)	92.2 (89.0, 94.3)	0.856
RDW-SD, Median (IQR)	44.1 (41.9, 46.6)	44.4 (41.3, 47.0)	0.714
NEUT%, Median (IQR)	85.5 (80.7, 89.6)	86.1 (80.5, 89.3)	0.572
LY%, Median (IQR)	7.8 (5.6, 11.4)	7.7 (5.5, 10.7)	0.568
EOS%, Median (IQR)	0.1 (0.0, 0.3)	0.1 (0.0, 0.3)	0.086
Fibrinogen, Median (IQR)	2.14 (1.62, 3.34)	2.17 (1.60, 3.03)	0.575
TBil, Median (IQR)	16.4 (11.6, 21.9)	16.8 (11.7, 22.7)	0.722
CysC, Median (IQR)	1.03 (0.82, 1.38)	1.02 (0.83, 1.39)	0.958
Ca^2+^, Median (IQR)	2.16 (2.06, 2.24)	2.15 (2.08, 2.24)	0.433
Mg^2+^, Median (IQR)	0.82 (0.77, 0.90)	0.81 (0.76, 0.88)	0.500
ALT, n (%)	32 (12.6)	18 (16.7)	0.321
AST, n (%)	39 (15.4)	22 (20.4)	0.283
LDH, n (%)	30 (11.9)	19 (17.6)	0.179

**Table 4:** Data centroid and weights of binary variables.

Variables	Centroid Coordinates	Weights *
Gender	0.107	−0.79
Chest Pain	0.953	0.91
Back Pain	0.865	0.73
Abdominal Pain	0.091	−0.82
Dyspnea	0.029	−0.94
Hypertension	0.851	0.70
β_1_-blocker	0.899	0.80
ACEI/ARB	0.220	−0.56
α-blocker	−0.100	−1.20
Morphine	1.048	1.10
Hydrochlorothiazide	−0.102	−1.20
ALT	0.045	−0.91
AST	0.052	−0.90
LDH	0.023	−0.95

* The weight was rounded to two decimal places.

**Table 5:** Data centroid and reference value of continuous variables.

Variables	Centroid Coordinates	Reference Value *
age	0.609	58.80
McV	0.677	92.77
RDW-SD	0.720	45.53
NEUT%	0.799	86.21
LY%	0.134	8.39
EOS%	0.016	0.13
fibrinogen	0.233	2.41
TBil	0.172	16.44
CysC	0.130	1.34
Ca^2+^	0.466	2.12
Mg^2+^	0.037	0.88

* The reference value corresponds to the actual value of the variables at the coordinate of the centroid, rounded to two decimal places.

**Table 6:** Confusion matrix of the test result of centroid regression.

	Positive Samples	Negative Samples
Predicted as positive samples	39	1
Predicted as negative samples	9	59

**Table 7:** Accuracy, sensitivity, specificity, Youden’s index, AUROC and AUPRC comparison between prediction results of centroid regression and other models.

	Centroid Regression	Logistic Regression	Classification and Regression Tree	Explainable Boosting Machine	Extreme Gradient Machine
Accuracy	90.7%	81.5%	93.6%	91.7%	95.4%
Sensitivity	0.813	0.741	0.891	0.913	0.957
Specificity	0.983	0.900	0.968	0.921	0.952
Youden’s index	0.796	0.641	0.860	0.834	0.909
AUROC	0.953	0.843	0.961	0.940	0.974
AUPRC	0.978	0.863	0.969	0.943	0.968

**Table 8:** Result of K-fold.

	Accuracy	Sensitivity	Specificity	Youden’s Index	AUROC	AUPRC	Size
1	91.9%	0.909	0.929	0.838	0.908	0.957	37
2	94.4%	0.905	0.942	0.847	0.937	0.969	36
3	97.2%	0.941	0.941	0.882	0.981	0.985	36
4	94.4%	0.952	0.933	0.885	0.987	0.991	36
5	86.1%	0.737	1.00	0.737	0.923	0.945	36
6	94.4%	0.950	0.938	0.888	0.981	0.986	36
7	88.9%	0.875	0.917	0.792	0.910	0.961	36
8	86.1%	0.826	0.923	0.749	0.916	0.959	36
9	91.7%	0.900	0.938	0.838	0.978	0.983	36
10	88.9%	0.818	1.00	0.818	0.951	0.974	36
Mean ± SD	0.914 ± 0.036	0.881 ± 0.065	0.946 ± 0.028	0.827 ± 0.051	0.947 ± 0.031	0.971 ± 0.014	
95% CI	(0.888, 0.940)	(0.835, 0.927)	(0.926, 0.966)	(0.790, 0.864)	(0.925, 0.969)	(0.961, 0.981)	

## Data Availability

Data is unavailable due to privacy or ethical restrictions.

## Abbreviations

The following abbreviations are used in this manuscript:

ACEI	Angiotensin-converting enzyme inhibitor
ALT	Alanine aminotransferase
ARB	Angiotensin II receptor blocker
AST	Aspartate aminotransferase
ATAAD	Acute type A aortic dissection
AUPRC	Area under the precision–recall curve
AUROC	Area under the receiver operating characteristic curve
CART	Classification and regression tree
CI	Confidence interval
CysC	Cystatin C
EBM	Explainable boosting machine
EOS	Eosinophil
LDH	Lactate dehydrogenase
LY	Lymphocyte
McV	Mean corpuscular volume
NEUT	Neutrophil
RDW-SD	Red cell distribution width–standard deviation
TBil	Total bilirubin
XGBoost	Extreme gradient boosting

## Appendix A

### Appendix A.1. Interpretability Analysis

The weights of binary variables could be calculated for global interpretability. The weight of the *i*-th binary variable w_i_ was defined as the difference in the feature value attributed to **X**_i_. The w_i_ is defined as follows:

(A1) $$w_{i}=d{|}_{X_{i}=0}-d{|}_{X_{i}=1}=2C_{i}-1$$

The weight shows the significance of the variables. If |w_i_| > |w_j_|(0 ≤ *i*, *j* < *m*), which shows the *i*-th variable is more significant on the mortality of ATAAD than the *j*-th variable. If w_i_ < 0, **X**_i_ = 0 is the risk factor of mortality of ATAAD. Otherwise, **X**_i_ = 1 is the risk factor.

The gradient of *d* (∇*d*) could be calculated as local interpretability. The process could be completed in linear complexity as follows:

(A2) $$\frac{\partial d}{\partial X_{j}}=2(X_{j}-C_{j})$$

(A3) $$\frac{dX_{j}}{dD_{j}}=(R_{j}-L_{j}{)}^{-1}\frac{dT_{j}(x)}{dx}{|}_{x=D_{j}}$$

Note that **D**_j_ is the raw data of the j-th continuous variable.

The ∇*d* shows the relation between the change in the patient’s mortality risk and each variable.

Centroid regression is a novel model designed for solving medical scenarios with high interpretability. Algorithm interpretability is obvious. Every step of the model can trace back to Formulas (1)–(8). Global interpretability aims to understand the overall behavior of a model and the influence of each feature on the result. The weight and significance of each variable are strongly associated, and the weight of variables (shown in Formula (A1)) is considered as the global interpretability of centroid regression. The local interpretability focuses on explaining the reasons for different results from different individuals. The local interpretability is much higher than black-box models such as decision trees, support vector machines, and deep neural networks, since the gradient of feature value *d* can be solved in linear complexity (Formulas (A2) and (A3)), and the feature value is directly linked to the mortality risk. The linear complexity has achieved the theoretical lower bound. However, the association between variables or variable combinations would not be well interpreted by centroid regression, especially for continuous variables. All the variables are considered as independent factors in centroid regression, but in clinical scenarios, correlation among variables is unavoidable (e.g., age and hypertension).

### Appendix A.2. Complexity Analysis

In one training process, the main time consumption is the Formula (5), with O(*nm*) time complexity, and it would run log*_κ_ε* times. Thus, the time complexity of one training process is O(*nm*log*_κ_ε*).

The testing process is traversing as Formula (8). Thus, the time complexity of testing for each sample is O(*m*).

There was no additional space consumption in the algorithm. Thus, the total space complexity is the space of patients’ data O(*nm*).

The time complexity represents the time to get the outcome from the algorithm and the space complexity represents the space occupation for running the algorithm. The time and space complexity of centroid regression are both low, especially the time complexity of testing, which lowers the threshold of clinical use.

The time complexity of testing and the total space complexity are both linear, and have achieved the theoretical lower bound. In the training process, the main time consumption is the inner product of vectors in the iteration process (Formula (5)), and the number of iterations is currently O(log), which is difficult to optimize. The aim of the training is to find the best centroid, or find the best point in an unordered space, and the theoretical lower bound of time complexity of the problem is the complexity of binary search, which is O(log). Thus, the time complexity of training is almost near the theoretical lower bound.
